# Prognostic and Predictive Model for Stage II Colon Cancer Patients With Nonemergent Surgery: Who Should Receive Adjuvant Chemotherapy?

**DOI:** 10.1097/MD.0000000000002190

**Published:** 2016-01-08

**Authors:** Chun-Dong Zhang, Ji-Nan Wang, Bai-Qiang Sui, Yong-Ji Zeng, Jun-Qing Chen, Dong-Qiu Dai

**Affiliations:** From the Department of Gastrointestinal Surgery, the Fourth Affiliated Hospital of China Medical University, Shenyang (C-DZ, B-QS, Y-JZ, D-QD); Department of General Surgery, Dalian Friendship Hospital, Dalian (J-NW); Cancer Center, the Fourth Affiliated Hospital of China Medical University (D-QD); and Cancer Research Institute, China Medical University, Shenyang, PR China (D-QD, J-QC).

## Abstract

No ideal prognostic model has been applied to clearly identify which suitable high-risk stage II colon cancer patients with negative margins undergoing nonemergent surgery should receive adjuvant chemotherapy routinely.

Clinicopathologic and prognostic data of 333 stage II colon cancer patients who underwent D2 or D3 lymphadenectomy during nonemergent surgery were retrospectively analyzed.

Four pathologically determined factors, including adjacent organ involvement (RR 2.831, *P* = 0.001), histologic differentiation (RR 2.151, *P* = 0.009), lymphovascular invasion (RR 4.043, *P* < 0.001), and number of lymph nodes retrieved (RR 2.161, *P* = 0.011), were identified as independent prognostic factors on multivariate analysis. Importantly, a simple cumulative scoring system clearly categorizing prognostic risk groups was generated: risk score = ∑ coefficient’ × status (AOI + histological differentiated + lymphovascular invasion + LNs retrieved).

Our new prognostic model may provide valuable information on the impact of lymphovascular invasion, as well as powerfully and reliably predicting prognosis and recurrence for this particular cohort of patients. This model may identify suitable patients with an R0 resection who should receive routine postoperative adjuvant therapy and may help clinicians to facilitate individualized treatment.

In this study, we aim to provide an ideal and quantifiable method for clinical decision making in the nonemergent surgical treatment of stage II colon cancer. Our prognostic and predictive model should be applied in multicenter, prospective studies with large sample sizes, in order to obtain a more reliable clinical recommendation.

## INTRODUCTION

Cancer has emerged as a major global public health problem, with 1 in 4 deaths in the United States attributed to this disease. A total of 102,480 new colon cancer cases and 50,830 colorectal cancers deaths were projected by the American Cancer Society to occur in the United States in 2013.^1^ Despite earlier diagnosis and advances in therapy, colorectal cancer remains the third most frequently diagnosed cancer and the fifth leading cause of cancer-related mortality in China. Generally, surgery is initially curative for stage I colon cancer; most stage III/IV patients benefit from adjuvant chemotherapy.^2–4^

Adjuvant chemotherapy remains controversial in stage II colon cancer, including who would benefit from adjuvant chemotherapy.^5,6^ However, identification of high-risk stage II colon cancer patients who would benefit from adjuvant treatment remains a clinical concern. The National Comprehensive Cancer Network (NCCN) Guidelines for colon cancer do not clearly distinguish patients who have a high risk of recurrence or who might benefit from adjuvant chemotherapy.^4^ Remarkably, these guidelines suggest that adjuvant chemotherapy should be considered for stage II patients with high-risk features, including T4 staging, poorly differentiated tumors, lymphovascular or perineural invasion, obstructing or perforating cancers, positive margins, and inadequately (<12) sampled lymph nodes (LNs).^4,7–12^ However, no details of recommendations on the use of adjuvant therapy in these situations are provided.^4^ On the contrary, observation without adjuvant therapy is also an option.^4^ Thus, the decision to offer adjuvant therapy remains unclear. Moreover, for stage II colon cancer patients with emergent features (obstruction, or localized or near-perforation), nonemergent surgery is preferable to emergent surgery.

Four pathologic factors (peritoneal involvement, venous spread, involved surgical margin, and perforation through the tumor) have been combined into a cumulative scoring system for prognostic assessment in patients with stage II colon cancer,^13^ which was also proven reliable for patients with stage II colorectal cancer.^14^ Most patients who undergo nonemergent surgery obtain negative margins, which addresses our concerns of determining a suitable prognostic model for these patients.

We aimed to evaluate different prognostic factors in order to design a predictive and prognostic model to identify suitable high-risk stage II colon cancer patients undergoing nonemergent surgery with a D2 or D3 lymphadenectomy, to receive adjuvant chemotherapy.

## METHODS

### Ethics Statement

This study was approved by the Ethics Committee of the Fourth Affiliated Hospital, China Medical University. All patient records and information were anonymized and deidentified prior to analysis.

### Included and Excluded Standards

Data of patients with stage II colon cancer were entered into a retrospective database between January 2003 and July 2012. These patients were treated at the Department of Gastrointestinal Surgery, the Fourth Affiliated Hospital of China Medical University, and the Cancer Research Institute of China Medical University. Overall, 402 patients with stage II colon cancer underwent D2 or D3 lymphadenectomy with negative resection margins (R0). Patients with perforation or obstruction who required emergent surgery had a worse prognosis compared to patients with nonemergent surgery;^15–18^ therefore, we only included patients without perforation or obstruction, giving a total of 333 patients enrolled.

The inclusion criteria were as follows: (1) histologically proven adenocarcinoma; (2) diagnosed as stage II cancer; (3) negative resection margins (R0); (4) potentially curable and curative operation was performed; (5) with nonemergent surgery; (6) a complete medical record was available; (7) with D2 or D3 lymphadenectomy. The exclusion criteria were as follows: (1) with emergent surgery (obstruction, or localized or near-perforation); (2) died in the postoperative period (<30 days postoperatively); (3) previous or concomitant other cancer; (4) lost to follow-up.

Of the 69 patients not included, 11 died in the postoperative period (<30 days postoperatively), 23 were lost to follow-up, 13 were underwent with emergent surgery because of perforation, and 22 underwent with emergent surgery because of obstruction.

### Definition of Stage II Colon Cancer

According to the current NCCN Guidelines for colon cancer,^4^ stage II disease, characterized by full thickness tumor invasion of the bowel wall and the absence of lymph node metastases, was subdivided into IIA (T3 lesions invading through the muscularis propria into the pericolorectal tissues), IIB (T4a lesions directly penetrating to the surface of the visceral peritoneum), and IIC (T4b lesions directly invading or adherent to other organs or structures), depending on where the primary tumor was located (T3 and T4). Moreover, lymphovascular invasion (LVI) was defined as present when tumor cells or tumor thrombi were found within an endothelium-lined space, or if the lymphovascular wall was destroyed by tumor cells.^19^

### Pathology Methods

The carcinoma lesions together with the surrounding bowel wall were fixed in formalin and cut into multiple 5 mm slices; 2 pathologists independently examined the sections and disagreements were resolved by discussion to determine the final diagnosis. The NCCN Guidelines recommended examination of a minimum of 12 LNs to accurately identify stage II colorectal cancers.^7,20,21^ If fewer than 12 LNs were initially identified, resubmission was performed in order to identify as many LNs as possible.

### Clinicopathologic Characteristics

The clinicopathologic features that were investigated for prognostic significance included age, gender, tumor size, tumor location, differentiation, LVI status, depth of penetration (pT), number of negative LNs retrieved, chemotherapy use, follow-up period, date of death, causes of death, and other factors (Table 1). Median and mean follow-up periods were 45 and 52.23 ± 29.7 months, respectively. Recurrence was classified as in situ recurrence, liver, peritoneum, bone, lung, and brain. All recurrences were diagnosed clinically or radiographically through contrast-enhanced computed tomography (CT) scans of the chest, abdomen, and pelvis every 3 months for the first 2 years, and every 6 to 12 months for a total of 5 to 6 years; head computed tomography, bone scan, and other diagnostic tests were performed if necessary.^22^ Furthermore, survival time was calculated from the date of surgery to the date of death or censoring (July 31, 2012).

**TABLE 1:**
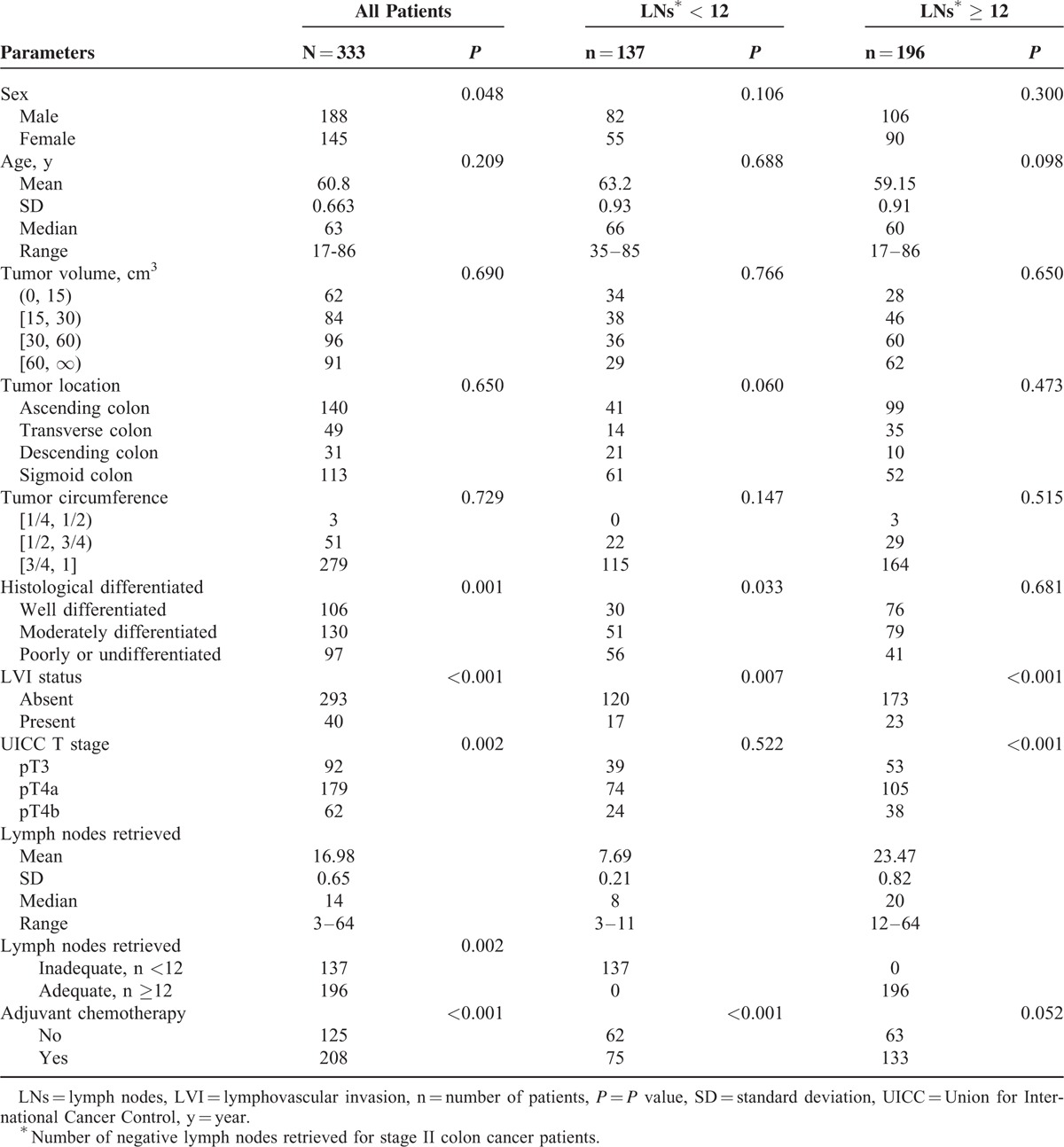
Univariate Analysis of Clinicopathologic Factors for Stage II Colon Cancer Patients

### Statistical Analysis

**FIGURE 1:**
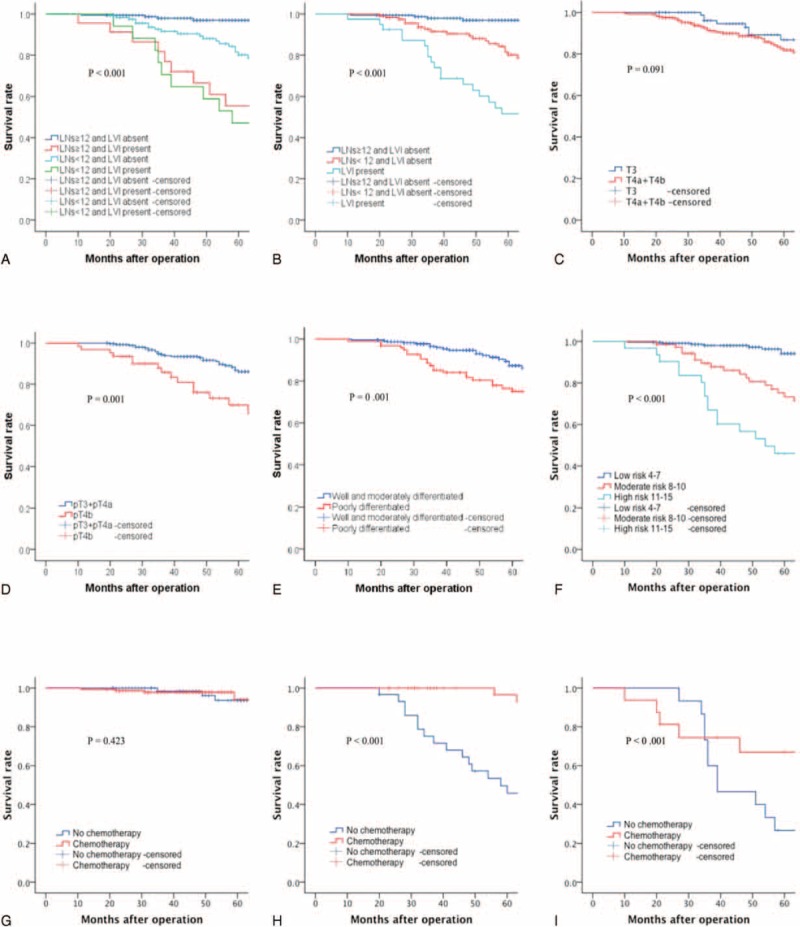
Kaplan–Meier overall survival analysis. (A) For stage II colon cancer patients according to LVI status and negative lymph nodes retrieved: lymph nodes ≥12 and LVI absent versus lymph nodes <12 and LVI absent (*P* < 0.001); lymph nodes <12 and LVI absent versus lymph nodes ≥12 and LVI present (*P* = 0.010); lymph nodes ≥12 and LVI present versus lymph nodes <12 and LVI present (*P* = 0.877) (overall effect: *P* < 0.001). (B) For stage II colon cancer patients according to LVI status and negative lymph nodes retrieved: lymph nodes ≥12 and LVI absent versus lymph nodes <12 and LVI absent (*P* < 0.001); lymph nodes <12 and LVI absent versus LVI present (*P* = 0.001) (overall effect: *P* < 0.001). (C) For stage II colon cancer patients according to the UICC pT stage: pT3 versus pT4a + pT4b (*P* = 0.091). (D) For stage II colon cancer patients according to adjacent organ involvement: absent (pT3 + pT4a) versus present (pT4b) (*P* = 0.001). (E) For stage II colon cancer patients according to the histological differentiated: well- and moderately differentiated versus poorly and undifferentiated (*P* = 0.001). (F) For stage II colon cancer patients according to the prognostic model: low risk versus moderate risk (*P* < 0.001), moderate risk versus high risk (*P* = 0.003) (overall effect: *P* < 0.001). (G) For low risk patients according to adjuvant chemotherapy: no chemotherapy versus chemotherapy (*P* = 0.423). (H) For moderate risk patients according to adjuvant chemotherapy: no chemotherapy versus chemotherapy (*P* < 0.001). (I) For high-risk patients according to adjuvant chemotherapy: no chemotherapy versus chemotherapy (*P* < 0.001). LVI = lymphovascular invasion, UICC = Union for International Cancer Control.

Rates of overall survival (OS) were obtained using Kaplan–Meier survival analysis (Figure 1). Univariate analysis and Cox's proportional hazard model in the multivariate analysis were conducted. The log-rank test was applied to select the optimum cutoff score according to the maximum chi-squared value. A *P* value of <0.05 was considered statistically significant. All statistical calculations were performed with SPSS software, version 19 (SPSS Inc, Chicago, IL).

## RESULTS

Clinicopathologic features and the univariate analysis of prognostic factors for patients with stage II colon cancer are listed in Table 1. A total of <12 LNs was considered inadequate, and ≥12 LNs was considered adequate. In the univariate analysis, LVI status (*P* < 0.001), pT classification (*P* = 0.002), LNs retrieved (*P* = 0.002), histologic differentiation (*P* = 0.001), and adjuvant chemotherapy (*P* < 0.001) were identified as significantly correlating with prognosis.

### Patient and Disease Characteristics

Of the remaining 333 stage II patients, 137 (41.1%) had < 12 LNs retrieved and 196 (58.9%) had ≥12 LNs retrieved, with an average of 16.98 ± 0.65 (range 3–64) LNs retrieved (7.69 ± 0.21 and 23.47 ± 0.82, respectively). Overall, 92 (27.6%), 179 (53.8%), and 62 (18.6%) patients were classified as stage IIA, IIB, and IIC, respectively.

### Formation of the Prognostic and Predictive Model

We undertook several steps to create our prognostic and predictive model. First, 6 factors, including sex (*P* = 0.048), degree of differentiation (*P* = 0.001), LVI status (*P* < 0.001), NCCN T stage (*P* = 0.002), LNs retrieved (*P* = 0.002), and adjuvant chemotherapy (*P* < 0.001) were found to be statistically significant (Table 1). Because this model was created to decide whether to offer adjuvant chemotherapy, the receipt of adjuvant chemotherapy itself should not be applied to this model. We also found that AOI status (pT3 and pT4a implied the absence of adjacent organ involvement while pT4b indicated adjacent organ involvement) was better for the model, compared with the NCCN T stage, which needs further verification.

Second, 4 variables, including AOI status (*P* = 0.001), differentiation (*P* = 0.009), LVI status (*P* < 0.001), and LNs retrieved (*P* = 0.011) were finally identified to be independent prognostic factors by the Cox regression model of multivariate analysis (Table 2) and the coefficients of 4 independent prognostic factors were identified by Cox regression of multivariate analysis. For easier calculation, we used coefficient’ instead of the coefficient (coefficient’ = 2.76 × coefficient). The score of the 4 variables (AOI status, differentiation, LVI status, and LNs retrieved) in the equation were 3, 2, 4, and 2, respectively (Table 2). Numeric variables included degree of differentiation and LNs retrieved. Differentiation was graded as 1 for well/moderately differentiated and 2 for poorly/undifferentiated. LN status was graded as 1 for ≥12 LNs retrieved and 2 for <12 LNs retrieved. Furthermore, dichotomous variables included AOI and LVI status. AOI was graded as 0 for absent and 1 for present. LVI was graded as 0 for absent and 1 for present (Table 2). Thus, the equation we designed for our prognostic model was as follows:

**TABLE 2:**
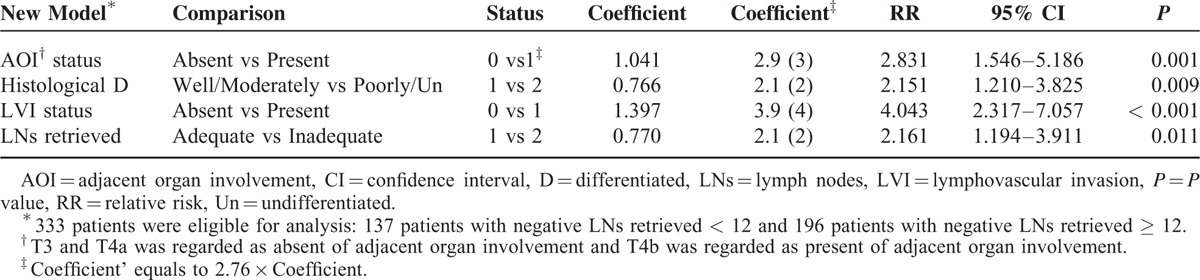
Multivariate Analysis of Prognostic Factors for Stage II Colon Cancer Patients

Risk score = ∑ coefficient’ × status (AOI + histological differentiated + lymphovascular invasion + LNs retrieved).

The risk score is 3 × AOI status + 2 × status of histologic differentiation + 4 × LVI status + 2 × status of LNs retrieved (Table 3). Accordingly, the risk score can have scores ranging from 4 to 15 (Table 3).

**TABLE 3:**
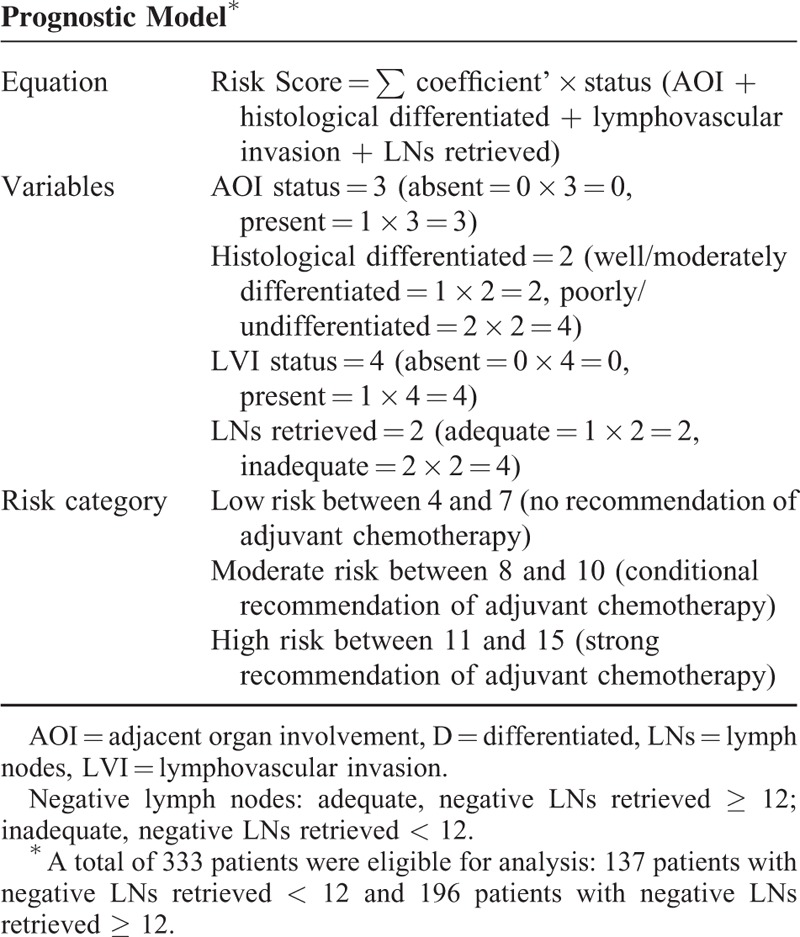
Prognostic Model for Stage II Colon Cancer Patients

Third, we calculated the risk score for each patient according to the individual status of prognostic factors. To find the optimal cutoff points, we conducted Kaplan–Meier curves of every score for these patients. The scores with the maximum chi-square values (or minimum *P* values) were the optimal cutoff points. Accordingly, we found 2 scores (7 and 10) with maximum chi-square values (44.19 and 49.93), whereas all the other chi-square scores were <40. Thus, patients could be divided into a low-risk group with a risk score of 4 to 7, a moderate-risk group with a risk score of 8 to 10, and a high-risk group with a risk score of 11 to 15 (Table 3). Furthermore, the Kaplan–Meier curve for low, moderate, and high-risk groups was demonstrated (*P* < 0.001; Figure 1F).

### Clinical Outcomes–Stage II Disease

The 5-year OS for stage II colon cancer patients is shown in Table 4. The 5-year OS for patients with inadequately retrieved LNs was lower than in patients with adequately retrieved LNs (stage IIA 76.8% vs 97.6%, stage IIB 74.6% vs 95.6%, respectively), proving that retrieved LNs were a poor prognostic factor for stage II patients (Table 4).

**TABLE 4:**
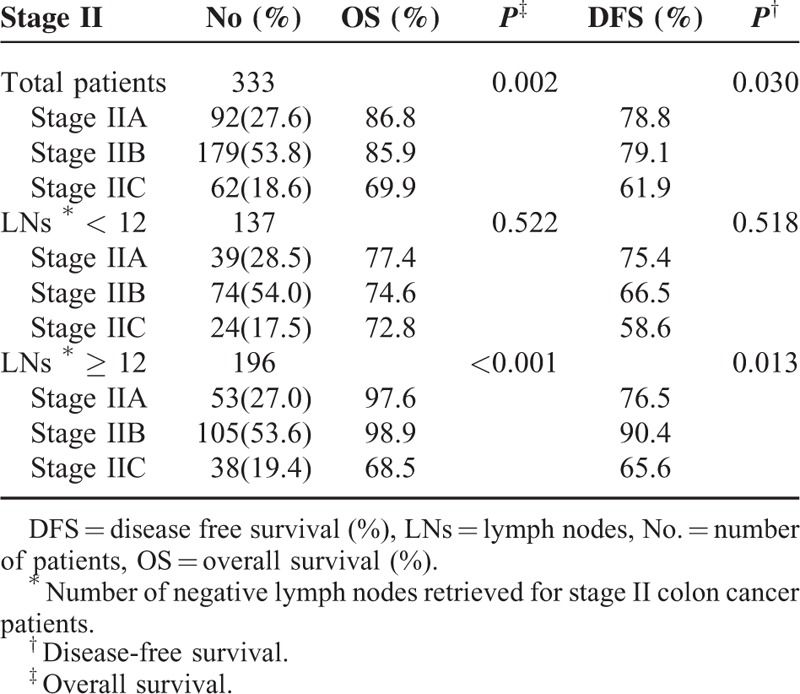
Five-Year Survival Rates of Stage II Colon Cancer Patients

### Clinical Outcomes–Recurrence and LVI

R0 was defined as complete tumor resection with all negative margins. Despite an R0 resection for all stage II colon cancer patients, recurrence was still observed (12.01%; 40 of the 333 recurred). Table 5 lists recurrence sites and causes of death for stage II colon cancer patients. As shown, 293 patients had absence of LVI (LVI−), whereas 40 had LVI present (LVI+). For patients with LVI−, the number of patients with <12 and ≥12 LNs retrieved was 120 and 173, respectively. Likewise, for patients with LVI+, the number of patients with <12 and ≥12 LNs retrieved was 17 and 23, respectively (Table 5).

**TABLE 5:**
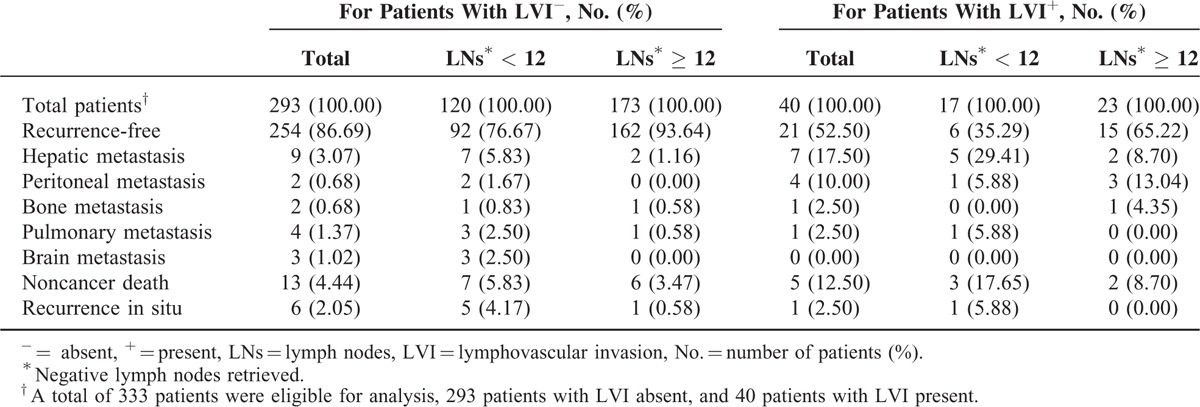
Recurrence Sites and Causes of Death for Stage II Colon Cancer Patients

## DISCUSSION

Generally, surgery with an R0 resection alone may be the most important treatment for resectable cancer initially, and patients will benefit from the improvement of the R0 resection rate. Therefore, we recommend that clinicians try to achieve an R0 resection for all patients. Concerning prognosis, a minimum of 12 negative LNs retrieved have been recommended by the NCCN Guidelines to accurately identify stage II colon cancer;^7,20,21^ however, the number of negative LNs retrieved can vary with age, gender, grade, and tumor site.^23^ As a result, some patients with stage II colon cancer still have <12 LNs retrieved; thus, not all stage II patients are correctly identified, which influences the application of adjuvant chemotherapy and patient surveillance.

The phenomenon of understaging due to insufficient LNs has been applied to represent the stage migration phenomenon,^24^ which should include 2 factors, including insufficient LNs dissected and insufficient LNs retrieved. In light of this consideration, surgeons should retrieve as many LNs as possible intraoperatively; D2 or D3 lymphadenectomy with R0 surgery should be achieved for stage II colon cancer patients. Also, pathologists should try to retrieve adequate LNs postoperatively, as recommended by NCCN Guidelines, as this has proven vital to improve the quality of pathologic reporting.^25^ Our further results support the observation that patients with stage II colon cancer benefit from adequate negative LNs retrieved compared to patients with inadequate negative LNs retrieved.

### Controversial Results of Adjuvant Chemotherapy in Stage II Disease

It remains controversial whether or not stage II colon cancer patients should receive adjuvant chemotherapy. An analysis of pooled data including 7 randomized controlled trials (RCTs) documented that stage II patients fail to benefit from 5-FU-based adjuvant chemotherapy.^5^ Similarly, an analysis of SEER databases showed no statistical significance when stage II patients receiving adjuvant chemotherapy, compared with those without adjuvant chemotherapy (HR 0.91, 95% CI 0.77–1.09).^6^ Moreover, another analysis from the SEER Medicare database showed no 5-year survival benefit for stage II patients with 1 or more poor prognostic features to receiving adjuvant chemotherapy, compared with observation (HR 1.03, 95% CI 0.94–1.13). On the contrary, the QUASAR trial suggested that patients with stage II disease benefited from adjuvant therapy of 5-FU/leucovorin (LV), compared to patients not receiving adjuvant therapy (RR 0.71, 95% CI 0.54–0.92, *P* = 0.01). Furthermore, a meta-analysis including 12 RCTs found a significantly improved survival for patients with stage II colon cancer (HR of 5-year OS 0.81, 95% CI 0.71–0.91); however, the chemotherapy regimens in these trials are not currently recommended.^26^ It is difficult for clinicians to decide whether or not patients with stage II colon cancer should receive adjuvant chemotherapy, especially when they have 1 or more high-risk features. Taking all these factors into consideration, we aimed to determine a suitable method to identify appropriate high-risk stage II colon cancer patients who should receive adjuvant chemotherapy.

### Individualized Management

We developed this new model to improve prognostication and prediction of recurrence for patients with stage II colon cancer. We found that it may be more suitable to apply AOI status (pT3 and pT4a as the absence of adjacent organ involvement and pT4b as the presence of adjacent organ involvement) into our model (Figure 1C and D), compared with the NCCN T stage. We will further investigate whether the NCCN T stage or AOI status is better for our model. Likewise, we combined well-differentiated and moderately differentiated tumors into 1 group, and poorly and undifferentiated into another group (Figure 1E). We found that this new model can clearly categorize patients into low-, moderate-, and high-risk groups, and demonstrated significant differences in survival (Figure 1F). Accordingly, patients in both high- and moderate-risk groups will benefit from adjuvant chemotherapy (Figure 1H and I), especially in the high-risk group; no survival benefit from adjuvant chemotherapy was observed in the low-risk group (Figure 1G). No adjuvant chemotherapy was recommended for the low-risk group (score between 4 and 7). Moreover, adjuvant chemotherapy was strongly recommended for the high-risk group (score between 11 and 15). Patients in the high-risk group should be considered for adjuvant chemotherapy with 5-FU/LV, capecitabine, FOLFOX, capecitabine/oxaliplatin (CapeOx), or FLOX, recommended by NCCN guidelines.^4,7,8^ Therefore, we recommended that patients in high-risk group should accept adjuvant chemotherapy; we suggested that patients in the moderate group accept adjuvant chemotherapy, or participate in clinical trials; follow-up was recommended for patients with the low-risk group.

In addition, we made a conditional recommendation for adjuvant chemotherapy in the moderate-risk group (score between 8 and 10; Table 3). For these patients, we recommend observation, and managing them by the CEA level, colonoscopy, CT scans, physical examination, and PET/CT scan every 3 months. If rising CEA levels and abnormal imaging results are observed, CEA level and imaging examinations will be obtained more frequently in order to reidentify high-risk patients, in order to add adjuvant chemotherapy. CEA testing and repeat CT scans are recommended every 3 months until the CEA level declines and stabilizes.^4^ We suggest that the “observation window” for monitoring should be at least 5 to 6 years. It is worthy to note that all patients who were identified as suitable patients for adjuvant chemotherapy should have a good tolerance, which refers to the Karnofsky performance score of ≥60% or the ECOG performance score of ≤2, and all patients requiring adjuvant chemotherapy should be no >70 years.^4,27,28^ Notably, clinicians should explain the prognosis clearly, and clinical decision marking should center on patient choice.^4,8^

### Lifestyle Care

In addition, patients were recommended to maintain healthy lifestyles postoperatively, including engaging in regular exercise (especially Tai Chi), maintaining a healthy body mass index (BMI), smoking and drinking cessation, and including a healthy diet of more fruits and vegetables, also recommended by NCCN Guidelines.^4^

### Clinical Implications

Concerning prognosis, we aimed to investigate the mechanism of why high-risk patients significantly benefit from adjuvant chemotherapy. High-risk patients may have a higher response rate to chemotherapy compared with the low-risk group. Adjuvant chemotherapy may also lower recurrence in high-risk patients, with a resulting survival benefit. Remarkably, in the high-risk group, the survival rate for patients with no adjuvant chemotherapy for 30 to 40 months significantly declined, demonstrating that CEA and radiographic tests should be used more frequently during this period (Figure 1I). We plan to perform further research with a larger sample size and in a multicenter format to confirm our hypothesis.

To further analyze the relationship between the number of negative LNs and LVI status, a Kaplan–Meier curve of the 4 groups, including ≥12 LNs retrieved with LVI–, <12 LNs retrieved with LVI–, ≥12 LNs retrieved with LVI+, and <12 LNs retrieved with LVI+, was performed in Figure 1A. As shown in patients with LVI–, survival significantly correlated with the number of negative LNs retrieved, which revealed that inadequately retrieved was a poor prognostic factor. However, for patients with LVI+, there was no statistical significance between negative LNs retrieved (≥12 and <12), which addressed our concerns. Furthermore, we combined the 2 groups of negative LNs retrieved, ≥12 with LVI+ and <12 with LVI+, and constructed another Kaplan–Meier curve (≥12 with LVI–, <12 with LVI– and LVI+; Figure 1B). A significant difference was observed based on the current limited evidences; we conjectured that the LVI status was a more important prognostic factor than the number of negative LNs retrieved and that patients with LVI+ had a worse prognosis than patients with LVI–, regardless of the number of negative LNs retrieved.

Another issue of concern is that few patients undergoing R0 resection with adequate negative LNs retrieved had developed peritoneal metastasis. Until now, there has been no universally accepted definition of micro-metastasis.^29–32^ However, the potential pathway of peritoneal metastasis is more of a concern. Patients with LVI+ were found to have a higher rate of peritoneal metastasis compared to patients with LVI– (OR 10.61, 95% CI 1.87–60.11, *P* = 0.008). Similarly, patients with pT4b had a higher rate of peritoneal metastasis compared to pT4a (OR 5.90, 95% CI 1.05–33.03, *P* = 0.04). Some patients were clinically diagnosed with tumor penetrating to the surface of the visceral peritoneum (pT4a), later proven to be inflammatory and penetrating according to postoperative pathology (pT3). Thus, we wanted to understand the potential pathways of peritoneal metastasis. First, the most likely pathway we conjectured was that LVI+ might result in LNs metastasis, with a few positive LNs involving the peritoneum. Second, tumor cells might invade the peritoneum directly; no patients with stage pT3 tumor had peritoneal metastasis and 2 patients with pT4a and 4 with pT4b stage developed peritoneal metastasis. Consequently, the mechanism for disease progression still needs to be validated.

Our results are limited by the retrospective study design and relatively small number of patients. Moreover, the period of the follow-up in some patients was <5 years, which may lead to bias and weaken our conclusion. Meanwhile, there were no unified criteria to decide whether or not to receive adjuvant chemotherapy; thus, selection bias may exist in this study. A propensity score model would go a long way and our prognostic model still needs to be validated in further studies. However, valuable information for the impact of LVI status may be found. This model may be powerful and reliable to predict prognosis and disease recurrence for patients with stage II colon cancer undergoing nonemergent surgery and may be applied to identify suitable patients with an R0 resection who should receive adjuvant therapy routinely. Furthermore, it may help clinicians to facilitate individualized management.

We have aimed to provide an ideal and quantifiable method for clinical decision making by identifying suitable patients for routine adjuvant chemotherapy. Our prognostic and predictive model requires testing in multicenter, prospective studies with large sample sizes in the future, in order to aid clinicians in making a better clinical recommendation. Whether our new model should be recommended for routine clinical application still requires further validation.
